# The cost of implementing the COVID-19 shielding policy in Wales

**DOI:** 10.1186/s12889-023-17169-3

**Published:** 2023-11-26

**Authors:** Bernadette Sewell, Angela Farr, Ashley Akbari, Andrew Carson-Stevens, Jeremy Dale, Adrian Edwards, Bridie Angela Evans, Ann John, Fatemeh Torabi, Stephen Jolles, Mark Kingston, Jane Lyons, Ronan A. Lyons, Alison Porter, Alan Watkins, Victoria Williams, Helen Snooks

**Affiliations:** 1 Swansea Centre for Health Economics, Faculty of Medicine, Health and Life Science, Swansea University, Singleton Park, Swansea, SA2 8PP UK; 2 Population Data Science, Faculty of Medicine, Health and Life Science, Swansea University, Singleton Park, Swansea, SA2 8PP UK; 3 PRIME Centre Wales, Division of Population Medicine, Cardiff University, Heath Park, Cardiff, CF14 4YS UK; 4 Warwick Medical School, University of Warwick, Coventry, CV4 7AL UK; 5 Swansea University Medical School and PRIME Centre Wales, Faculty of Medicine, Health and Life Science, Swansea University, Singleton Park, Swansea, SA2 8PP UK; 6 Immunodeficiency Centre for Wales, University Hospital of Wales, Cardiff, CF14 4XW UK; 7 Swansea University Medical School, Faculty of Medicine, Health and Life Science, Swansea University, Singleton Park, Swansea, SA2 8PP UK

**Keywords:** COVID-19, Shielding, Clinically vulnerable, Secure anonymised data, Implementation, Resources, Cost

## Abstract

**Background:**

The EVITE Immunity study investigated the effects of shielding Clinically Extremely Vulnerable (CEV) people during the COVID-19 pandemic on health outcomes and healthcare costs in Wales, United Kingdom, to help prepare for future pandemics. Shielding was intended to protect those at highest risk of serious harm from COVID-19. We report the cost of implementing shielding in Wales.

**Methods:**

The number of people shielding was extracted from the Secure Anonymised Information Linkage Databank. Resources supporting shielding between March and June 2020 were mapped using published reports, web pages, freedom of information requests to Welsh Government and personal communications (e.g. with the office of the Chief Medical Officer for Wales).

**Results:**

At the beginning of shielding, 117,415 people were on the shielding list. The total additional cost to support those advised to stay home during the initial 14 weeks of the pandemic was £13,307,654 (£113 per person shielded). This included the new resources required to compile the shielding list, inform CEV people of the shielding intervention and provide medicine and food deliveries. The list was adjusted weekly over the 3-month period (130,000 people identified by June 2020). Therefore the cost per person shielded lies between £102 and £113 per person.

**Conclusion:**

This is the first evaluation of the cost of the measures put in place to support those identified to shield in Wales. However, no data on opportunity cost was available. The true costs of shielding including its budget impact and opportunity costs need to be investigated to decide whether shielding is a worthwhile policy for future health emergencies.

## Background

The United Kingdom (UK) introduced the “shielding intervention” in March 2020 in response to the increased risk of morbidity and mortality following severe acute respiratory syndrome coronavirus 2 infection in Clinically Extremely Vulnerable (CEV) individuals. This included people suffering from respiratory disease, cancer or diabetes, and people receiving immunosuppressant medications [1]. The intervention lasted 10 months over two time periods (waves), ending in the spring of 2021.

In addition to introducing “lockdown” measures for the general population to help contain the spread of the virus, centralised routine National Health Service (NHS) data sources, algorithms and individual clinical screening of primary and secondary care records were used to compile a list of CEV people for shielding [2, 3]. Individuals on this shielding list were strongly advised by a series of letters, text messages or phone calls to self-isolate and to avoid contact with other people wherever possible, including within the home for initially 14 weeks. Food parcels, prescription delivery, and priority supermarket shopping and delivery slots were provided for people who had no support network. Furthermore, individuals who could not undertake their work without leaving their home were eligible for Statutory Sick Pay and the furlough scheme (paying 80% of wages for most people) [4].

Evidence of the effects of shielding on mental health, quality of life, social isolation, access to planned and emergency health care and mortality has now emerged [5–10]. However, the effects on COVID-19 infection rates and potential benefits are still being evaluated, and the question of whether this novel response to an unprecedented situation provided good value for money remains unanswered.

This paper reports the evaluation of the costs of implementing the shielding policy in Wales, UK, to the NHS and local government during the first wave of shielding (from March to June 2020). This cost analysis was undertaken as part of the first stage of the evaluation of the costs and consequences of the shielding support programme in Wales for the ‘EVITE Immunity’ study. This study aimed to evaluate the costs of the shielding policy and its effects on deaths, hospital and intensive care admissions and COVID-19 infections in the population identified for shielding in Stage 1; and to compared these outcomes to a similarly vulnerable group of non-shielded people using anonymised individual-level, population-scale routinely-collected data sources available within the Secure Anonymised Information Linkage (SAIL) Databank [11] trusted research environment (TRE) in Stage 2. Stage 2 of the study also investigates the impact of shielding status on immunity, safety, health-related quality of life, anxiety, depression, and loneliness [12].

## Methods

### The study population

The number of people identified for shielding in Wales was extracted from the COVID-19 Shielded People (CVSP) list data source within the SAIL Databank [13], which was provided from Digital Health and Care Wales (DHCW) based on the agreed methodology for those identified to shield [14]. In response to the outbreak of COVID-19, the so-called C20 Cohort was created to provide a population-level, electronic data resource to facilitate research assessing the impact of the COVID-19 pandemic [11]. The CVSP data within SAIL was used to identify persons in the C20 cohort who were formally recognised as shielded persons. The shielded population used in this study includes persons: i) identified on the CVSP list ii) with a valid date of being added to the list iii), added during 2020 [the enrolment period] iv) without a death date earlier than 23rd March 2020 and v) with a corresponding record in the C20 Cohort. A more detailed account of the methods of data extraction from SAIL can be found elsewhere [15]. Costs related to implementing the shielding policy in Wales were only available for the whole population supported. A breakdown of the costs for different cohorts with specific conditions was not available.

### The resource components of the shielding intervention

The resource components of the shielding intervention for CEV people in Wales during the early phase of the COVID-19 pandemic were mapped using a public sector perspective and based on published reports prepared by Welsh Government [16]. Documents and web pages relating to the management of COVID-19 were searched for relevant information regarding strategies and advice put in place to support people who were categorised as vulnerable and advised to stay at home for an initial 14 weeks period between March and June 2020. Where no published information on service provision or associated costs were available, freedom of information (FOI) requests were submitted to Welsh Government, and personal communications (e.g. with the office of the Chief Medical Officer for Wales and Digital Health Care Wales staff) were used to fill information gaps where required.

Based upon a logic model of the shielding programme developed by our study team in an earlier phase of work for the EVITE programme [17], four main components of the shielding intervention were identified, and unit costs were assigned as follows.

### Component 1: Costs of compiling the Shielded Patient List

The CVSP list was created by Digital Health and Care Wales (formerly NHS Wales Informatics Service, NWIS) with additional staff hours provided by the NHS Wales Delivery Unit (DU) and advice from Public Health Wales [14]. These central NHS bodies carried out two rounds of additions to the Welsh Shielded Patient List, with General Practitioners (GPs) identifying nearly 13,000 further patients.

Secondary care clinicians could also inform GPs if they felt a patient should be added to the list. However, some secondary care clinicians may have written to patients directly, and as a result these patients may not have been added to the Shielded Patient List [18]. From 25th May 2020, the Chief Medical Officer (CMO) for Wales instructed secondary care providers that any patients identified by secondary care would be collated by their health boards before being sent to Digital Health and Care Wales for checking and then letters issued centrally. GPs continued to provide their updated patient lists on a weekly basis as previously. By May 2020, approximately 130,000 patients had been identified for shielding across Wales [18].

Email and online audio-visual interviews with staff of the relevant organisations and the Office of the CMO for Wales were used to estimate the cost involved in this initial component of the shielding intervention.

### Component 2: Costs of contacting people advised to shield

The NHS Wales Shared Services Partnership (NWSSP) provided administrative support in managing the mail out of shielding letters between March and June 2020 to those identified for shielding. The first letter was sent on behalf of the CMO for Wales on 24th March 2020. This was followed up with a letter from local authority councils one to two weeks later and another letter on behalf of the CMO for Wales in June 2020. Due to the lack of available information, it was assumed that every person on the CVSP list was sent all three letters. Administrator time to handle the mail out (printing of letters, collating and folding into envelopes) was estimated to take two minutes per letter. Unit costs for letter materials were obtained online from an independent office supplier with staff time costed based on hourly rates for band 3 and 4 administrative roles [19].

### Component 3: Costs of pharmacy deliveries

Welsh Government provided two schemes to support medicines delivery to those who were identified for shielding during the first wave of the pandemic - the National Volunteer Prescription Delivery Scheme and the Royal Mail Track 24 Click and Drop Scheme [20]. Freedom of information requests were submitted to Welsh Government to obtain information on the cost of these schemes [21, 22].

#### The National Prescription Delivery Scheme

The National Volunteer Prescription Delivery Scheme was announced by the Welsh Government’s Minister for Health and Social Care on 5th May 2020. Volunteers were recruited by the British Red Cross and St John Ambulance Cymru. Furthermore, Welsh Government officials recruited volunteers from staff who were on furlough through liaison with pharmaceutical manufacturers, Optometry Wales, the Driving and Vehicle Standards Agency, and various Welsh Government departments. Moreover, individuals from public sector organisations whose routine duties were reduced and who had appropriate Disclosure and Barring Service clearance, could contact Welsh Government directly to volunteer.

#### Cost of Pro Delivery Manager software

As part of the National Prescription Delivery Scheme, a logistics software package called Pro Delivery Manager (PDM) was installed in community pharmacies and dispensing general practices in Wales to support delivery route planning and scheduling for medicines deliveries [23].

#### Royal Mail Track 24 Click and Drop Scheme

In May 2020, Welsh Government also put in place a Royal Mail scheme to support patients for whom deliveries via the National Prescription Delivery Scheme were not possible. The Royal Mail commercial service delivered items via its ‘Tracked 24’ service, with postal delivery workers collecting prescription medicines from local pharmacies and dispensing general practices and delivering to patients the next day. The cost of this service was obtained through a freedom of information request [21].

### Funding for dispensing prescription items

From April 2020, an additional fee of 7.4p per item dispensed was payable to all pharmacies and dispensing general practices that confirmed to their health board that they had arrangements in place to support patients who shielded with no other means of collecting medicines [24]. This funding applied to all items dispensed and was re-purposed from the ‘global sum’, which are core contract payments to pharmacies and general practices. These payments would have been previously used to provide Medicines Use Reviews which were suspended during the pandemic (personal communication via email with Community Pharmacy Wales dated 16 04 2021). Data extracted from the SAIL Databank via the Welsh Dispensing Data Set (WDDS) [25] were used to estimate the number of recorded dispensed prescriptions during the 14-weeks shielding period between April and June 2020 for people in the CVSP list.

### Component 4: Free food box scheme via local authorities

At the beginning of April 2020, Welsh Government launched a national food box scheme [26]. This was provided for shielded individuals without family or friends nearby, or without access to the internet or the means to pay for food. These funds were intended to supply food parcels, designed to support one person nutritionally for one week, over the initial shielding period. The funds made available were based on an estimated 30% uptake of the scheme. Costs of the food box scheme were provided by Welsh Government through a freedom of information request [27].

Alongside the free food boxes, priority food delivery slots were made available to shielded people by eight major supermarket chains in Wales who had the capacity to offer home deliveries. These priority supermarket food delivery slots were not available immediately at the start of lockdown but became widely available during the middle of the shielding period taking the pressure off Welsh Government’s food box scheme. This enabled those who were identified for shielding and had the means and finances to order food themselves without having to leave their homes. Any costs incurred by private sector supermarkets to enable this scheme are excluded from this evaluation.

### Research management and public involvement

The EVITE Immunity research team included clinical, policy, academic, methodological and public contributor experts who had equal responsibility in all decisions to develop, manage and deliver this study. Two public contributors were co-applicants and members of the Research Management Group, and worked with six more individuals providing wider public input via a Patient Advisory Panel. An independent Study Steering Committee included two public contributors. Our public contributors and some academic co-applicants were personally directly or indirectly affected by implementation of the shielding policy [28, 29].

## Results

This study’s shielded population available within the SAIL Databank consisted of 117,415 CEV individuals in Wales between March and May 2020. However, by the end of the study period approximately 130,000 people were estimated to be on the Shielded Patient List (but may not have complete records in the SAIL Databank). In order to facilitate implementation of the shielding policy and the required support for people identified as CEV and advised to shield, the following costs were incurred and are summarised in Table 1.

### Costs of identifying CEV individuals required to shield

According to personal email communication with the office of the Chief Medical Officer (CMO) for Wales (dated 06 05 2021), no specific additional funding was made available to any of the organisations involved to facilitate the work they were asked to do to develop, compile and maintain the Shielded Patient List. Instead, existing staff were redeployed to the task. However, the number of staff hours required was not available or could not be compiled.

### Costs of contacting people advised to shield

The cost of the first mail shot letter (nine pages) was estimated to be £1.35 per person, equating to £158,698 for the whole shielded population sample of 117,415 people (see Table 1). The subsequent letters cost approximately £1.30 per letter (£152,323 per mail-out) with at a total cost of £463,344 for the three letters sent to patients during the initial 3-months shielding period, assuming all identified patients received all three letters.

### Costs of pharmacy deliveries

#### National Prescription Delivery Scheme: volunteer costs

No evidence was available to estimate the number of volunteers involved in the National Volunteer Prescription Delivery Scheme or time spent volunteering. Volunteers gave their own time free of charge to support the scheme. They were given permission from their employers to volunteer whilst furloughed or temporarily unable to perform their substantive roles within their organisations due to COVID-19 restrictions. As such, no additional funding was provided for recruits.

Recruitment and management of volunteers by the British Red Cross and St John Ambulance Cymru was agreed on a cost per site supported (community pharmacy/dispensing doctor premises) as part of the scheme [22]. For services provided between 1st April and 30th June 2020, £8,000 was paid to the British Red Cross. A small number of Disclosure and Barring Service (DBS) checks were funded for volunteers who did not have appropriate vetting status and whose support was required to cover areas where recruitment of volunteers was more difficult. These costs totalled £406. In total, 310 volunteers were recruited by 29th June 2020 (from the start of the pilot on 29th April). Mileage expenses claims were submitted by the British Red Cross for the period 1st April to 30th June 2020 totalling £4,500. Table 1 shows that total costs associated with volunteer recruitment and management which were £12,906.

#### Pro Delivery Manager software costs

The logistics software package known as Pro Delivery Manager (PDM) was made available to all community pharmacies and dispensing doctor sites who expressed an interest in being part of the scheme. License fees were funded for a total of 693 sites at a cost of £61,966.68, although only 375 sites were reported to actively use the service.

#### Royal Mail prescription delivery

In May and June 2020, Royal Mail delivered 514 prescriptions to those who were categorised as clinically vulnerable and strongly advised to shield, at a cost of £1,693.

### Dispensing prescriptions payment

SAIL data revealed that 99.2% of the population sample (*n* = 116,614) had at least one item dispensed during the study period and 2,475,025 items were dispensed to the entire shielded population over the three months at a total cost of £183,152.

### Cost of food boxes

The number of food boxes distributed across Wales via the national scheme was 193,896, with an additional 20,815 boxes distributed through local pilots (in Ceredigion and Carmarthen) and 4,500 through food banks. In total, 219,211 food boxes were delivered at a standard cost of £27.76 per box (although box content and cost varied due to availability of stock in each region). Based on the assumption that 30% of the identified shielded population required free food boxes, this would equate to 35,263 people receiving on average 6 boxes per person during the 12-week period. The essential food supply boxes were assembled by private sector catering companies and conveyed predominately through van and lorry deliveries undertaken by private companies and local authorities. The overall food box supply costs reported were £11,797,962.48 for the Wales National Food Box Scheme and £730,494.26 for local pilots and food banks. Taking into account £2,162.00 for printing (boxes and certificates), £44,000.00 programme delivery fees and £9,974.00 for research and evaluation, Welsh Government reports a total spend of £12,584,593.

**Table 1:** Implementation cost of the shielding intervention

	Resources required	Unit cost of materials/labour	Study population at start of shielding period	% of population	Total Cost
**Identification**	Identifying people for the shielding list: multi-agency task			100	No additional funds
**Letters**	1st class postage (3 mail shots)	0.85	117,415	100	£99,802.75*3 £299,408.25
	Envelope (A5 pocket) for 1st mail shot Envelope (standard wallet) * 2 mail shots	0.0387 0.0224	117,415	100	£4,543.96 £2,630.10 £2,630.10
	1st 9 page A4 letter 2nd 3 page A4 letter 3rd 3 page A4 letter	0.0569 0.0189	117,415	100	£6,680.91 £2,219.14 £2,219.14
	Admin costs of managing mail out (3 mail shots)	0.406	117,415	100	£47,670.49*3 £143,011.47
	**Total Cost of shielding letters**				**£463,343.07**
**Community Pharmacy costs**	**1st April to 30th June**: Volunteers delivering medication via the National Prescription Delivery Scheme: Volunteer recruitment costs (including DBS checks) Volunteer expenses	Data not available.		n/a n/a	£ not available £8,406 £4,500 **£12,906.00**
	**29th April to 30th June**: Pro Delivery Manager Software		Total licensed accounts created: 693	n/a	**£61,966.68**
	**1st May to 30th June**: Royal Mail via its ‘Tracked 24’ service:	n/a	514 deliveries	n/a	**£1,693.00**
	**1st April onwards**: Fee of 7.4p per prescription item dispensed	0.074	Total items dispensed over 3 months = 2,475,025	*n* = 116,614 (99.2%)	£183,152.00
**Free food parcels via Local Authority Councils**	Printing (boxes and certs) Programme Delivery fees Local Pilots (Ceredigion and Carmarthen LAs) Wales National Scheme Research and Evaluation		Total number of food boxes distributed in Wales 219,211		£2,162.00 £44,000.00 £730,494.26 £11,797,962.48 £9,974.00 **£12,584,593.00**
	From mid-March to end April there were daily meetings between the Shielded and Vulnerable People team in Welsh Government and WLGA and WCVA: opportunity cost				£0
**Total Cost associated with shielded people in Wales (requiring new funding)** (including the 7.4p prescription item cost which was not new funding)					**£13,124,501.00** (£13,307,653.75)
**Cost per shielded person (*n* = 117,415)**					**£112** £113 with 7.4p prescription item cost

## Discussion

This evaluation estimated the additional costs associated with shielding clinically extremely vulnerable (CEV) people in Wales, UK between March and May 2020. At the start of the shielding period, 117,415 CEV people were strongly advised to stay at home and avoid contact with other people and were supported through Welsh Government and local authority schemes at a total additional cost of £13,307,654 or £113 per person shielded. This included new resources required to inform CEV people of the shielding intervention and to support shielded people with prescription and food deliveries but excludes the cost of identifying people to include on the Shielded People List as no new funds were made available. Since the number of people on the Shielded Patient List varied as people were added or removed weekly with a final shielded population number at the end of the study period of approximately 130,000, the cost per person shielded is likely to lie between £102 and £113. The retrospective analysis of the demographic and clinical data available from the SAIL databank undertaken as part of the EVITE Immunity study found that the main proportion of the shielded population suffered from severe respiratory conditions, underwent immunosuppressive therapy or had cancer [15]. However, based on the available cost data, no breakdown of costs for different health conditions could be made to better understand the cost of shielding for different patient sub-groups.

To our knowledge, this is the first investigation of the cost of the shielding policy in the UK. Our evaluation draws upon information provided by Welsh Government and participating organisations within Wales and provides a comprehensive estimation of the direct new public sector costs associated with implementation of the shielding policy during its initial phase, over the first few months of the COVID-19 pandemic in 2020. It provides valuable insights into the additional resources required to deliver the shielding intervention. However, the question remains whether these additional resources were used in a cost-effective way. The shielding initiative was aimed at protecting the most vulnerable members of society from COVID-19 infection and reduce COVID-related mortality and morbidity in this population [30]. However, no clear impact of shielding on COVID-19 infection rate was found in the clinical part of Stage 1 of the EVITE Immunity study [15] with a similar proportion of the shielded population testing positive for COVID-19 compared to the general population. Stage 2 of the EVITE Immunity study, which us currently ongoing, compares the health outcomes and healthcare resource use and costs of the shielded population with a matched non-shielded cohort. It is hoped that this evaluation will shed more light on the effectiveness and cost-effectiveness of the shielding initiative in Wales, and its impact on mortality and morbidity in the most vulnerable in due course.

While the current evaluation represents the most complete estimation of additional costs required for managing, maintaining and supporting the shielding intervention in Wales, several assumptions had to be made that may affect the results. Furthermore, not all people on the Shielded Patient List may have received all three shielding letters which may affect the cost of informing shielded people of the policy included in this cost analysis.

Lastly, while the study succeeded in compiling the actual new costs and funds made available for this initiative, it was not possible to account for the opportunity costs of people volunteering or being redeployed from other tasks. This was due to the fact that much of the work involved in designing, managing and administering the scheme was “soaked up” within organisations, displacing other activities with no or limited records of the actual time and resources involved. It was therefore not possible to estimate the opportunity cost incurred by the shielding intervention. The production and collation of the Shielded Patient List was an evolving process during the first three months of the pandemic with multiple NHS agencies involved and was a complex process which was supported by redeployed public sector staff and not funded by new money. With regards to the volunteers signed up to the National Prescription Delivery Scheme, these were people from the public sector (e.g. allied health care professionals) whose roles were suspended and who would have been furloughed and so would not have incurred a loss of productivity. The food box scheme was undertaken by the 22 Local Authority (LA) councils on behalf of Welsh Government with redeployed staff. There is potential for 22 different approaches to administering this scheme as well as redeploying different mixes of staff that were available to each LA council and variable time taken for a LA with a more rural population compared to urban centres. Information for the opportunity costs could therefore not be compiled. Estimating the important, and potentially large opportunity cost when (and if) data become available in the future would allow a more complete picture of the true cost of the shielding policy/intervention.

Furthermore, this evaluation took the perspective of Welsh Government and NHS Wales. Therefore, any costs incurred and resources used by family members and carers to support people who were advised to shield was not taken into account.

Another limitation is that some new resources and costs could not be estimated. For example, secondary hospital services had to adapt their model of care of some people strongly advised to shield who received regular treatment for their health conditions. There appears to have been wide variation in the way secondary care teams adapted the way they delivered treatment such as going out to patients’ homes, changing clinic sites to private sector hospitals and managing the flow of shielded patients. This may well have had a cost impact with the need to retrain staff in new methods of delivering treatment and by reducing the number of patients receiving treatment per day with staff travelling to patients rather than patients attending clinic slots. However, no records were available to gauge the cost of these activities.

The shielding intervention was born from a rapid response to an emergency situation and required new funding as well as the redeployment of existing resources. Considering the cost of shielding in terms of both its actual budget impact and potentially substantial opportunity cost, the intended as well as unintended effects and consequences of shielding need to be carefully investigated and weighed up to be able to decide whether shielding as a policy for future health emergencies is a worthwhile endeavour.

## Conclusion

### Total additional cost of the shielding intervention

Taking into account the new resources required to inform CEV people of the shielding intervention and support shielded people with prescription and food deliveries, and based on the population number at the beginning of the shielding period of 117,415 the total additional cost of the shielding intervention etc. was £13,307,654 or £113 per person shielded. However, the number of people on the Shielded Patient List varied as people were added or removed weekly. Using the population number at the end of the study period of 130,000, the total additional cost would be £102 per person. The cost per person shielded therefore lies between £102 and £113 as the Shielded Persons list was adjusted over the three month period.

## Data Availability

The cost data analysed during this study are included in this published article. Other data used in this study are available in the SAIL Databank at Swansea University, Swansea, UK. All proposals to use SAIL data are subject to review by an independent Information Governance Review Panel (IGRP).

## Abbreviations

CEV: Clinically extremely vulnerable
NHS: National Health Service
CVSP: COVID-19 Shielded People
DHCW: Digital Health and Care Wales
FOI: Freedom of information
NWIS: NHS Wales Informatics Service
DU: NHS Wales Delivery Unit
GPs: General Practitioners
CMO: Chief Medical Officer
NWSSP: NHS Wales Shared Services Partnership
PDM: Pro Delivery Manager
WDDS: Welsh Dispensing Data Set
DBS: Disclosure and Barring Service
